# The evolutionary genetics of lactase persistence in seven ethnic groups across the Iranian plateau

**DOI:** 10.1186/s40246-019-0195-5

**Published:** 2019-02-11

**Authors:** Hadi Charati, Min-Sheng Peng, Wei Chen, Xing-Yan Yang, Roghayeh Jabbari Ori, Mohsen Aghajanpour-Mir, Ali Esmailizadeh, Ya-Ping Zhang

**Affiliations:** 1 State Key Laboratory of Genetic Resources and Evolution, Kunming Institute of Zoology, Chinese Academy of Sciences, Kunming, 650223 China; 2 Kunming College of Life Science, University of Chinese Academy of Sciences, Kunming, 650204 China; 3 KIZ-CUHK Joint Laboratory of Bioresources and Molecular Research in Common Diseases, Kunming Institute of Zoology, Chinese Academy of Sciences, Kunming, 650223 China; 4 Biological Big Data College, Yunnan Agricultural University, Kunming, 650201 China; 5 State Key Laboratory for Conservation and Utilization of Bio-resources, Yunnan University, Kunming, 650091 China; 6 Faculty of Agriculture, Shahid Bahonar University of Kerman, Kerman, PB 76169-133 Iran; 7 Department of Genetics, Faculty of Medicine, Babol University of Medical Sciences, Babol, 4719173716 Iran; 8 Department of Medical Genetics, School of Medicine, Tehran University of Medical Sciences, Tehran, 14155-6447 Iran; 9 Center for Excellence in Animal Evolution and Genetics, Chinese Academy of Sciences, Kunming, 650223 China

**Keywords:** Lactase persistence, Iran, − 13910*T, − 22018*A, − 13915*G, Selection

## Abstract

**Background:**

The ability to digest dietary lactose is associated with lactase persistence (LP) in the intestinal lumen in human. The genetic basis of LP has been investigated in many populations in the world. Iran has a long history of pastoralism and the daily consumption of dairy products; thus, we aim to assess how LP has evolved in the Iranian population. We recruited 400 adult individuals from seven Iranian ethnic groups, from whom we investigated their lactose tolerance and screened the genetic variants in their lactase gene locus.

**Results:**

The LP frequency distribution ranged from 0 to 29.9% in the seven Iranian ethnic groups with an average value of 9.8%. The variants, − 13910*T and − 22018*A, were significantly associated with LP phenotype in Iranians. We found no evidence of hard selective sweep for − 13910*T and − 22018*A in Persians, the largest ethnic group of Iran. The extremely low frequency of − 13915*G in the Iranian population challenged the view that LP distribution in Iran resulted from the demic diffusion, especially mediated by the spread of Islam, from the Arabian Peninsula.

**Conclusions:**

Our results indicate the distribution of LP in seven ethnic groups across the Iranian plateau. Soft selective sweep rather than hard selective sweep played a substantial role in the evolution of LP in Iranian populations.

**Electronic supplementary material:**

The online version of this article (10.1186/s40246-019-0195-5) contains supplementary material, which is available to authorized users.

## Background

Lactase persistence (LP; OMIM #223100) is defined as the continued lactase enzyme activity that helps to digest lactose in dairy products in human adulthood [1]. It follows a Mendelian autosomal heritance [2] regulated by *cis*-acting elements of the lactase gene (*LCT*; OMIM *603202) [3]. Series of studies revealed five regulatory variants that are located in the 14 kb upstream of *LCT* in various populations: − 13910*T (rs4988235) in Europeans [4], Central Asians [5], and South Asians [6]; − 13915*G (rs41380347) in West Asians [7]; and − 13907*G (rs41525747), − 14009*G (rs869051967), and − 14010*C (rs145946881) in East Africans [8–10]. In addition, − 22018*A (rs182549) was investigated as a LP-associated variant [4, 11] in several populations [12–14], but it directed minimal enhancement of *LCT* promoter activity in vitro [15, 16]. The existence of independent variants underlying LP in different populations presents a paradigm of convergent evolution potentially driven by the daily consumption of large amounts of milk products after the domestication of dairy animals [17]. The co-evolution of genes for LP and milk consumption also becomes one of the most well-known gene-culture models for human evolutionary change [18, 19]. It also attracts wide interests from Neolithic archeology [20], in that the LP variant − 13910*T serves as a genetic marker in ancient DNA analyses to trace prehistoric migrations in Europe [21–23].

The genetic and archeological evidence supports the role of Iran as a domestication center of dairy animals such as goat [24, 25], cattle [26], and camel [27, 28]. The domesticated water buffaloes are also kept for milk production in Iran [29]. The long history of pastoralism and milk consumption raise the interest to explore the LP distribution as well as its genetic evolution in Iran. An early investigation of lactose intolerance revealed that the percentage of LP was 14% in Iranian adults [30]. The recent genetic screening for Iranians showed the occurrence of − 13910*T at 10% [7, 31]. The genotype-phenotype correlation was high [32], suggesting that − 13910*T might explain LP in Iranian population [33]. Despite these results, only limited samples from 42 individuals were analyzed so far. Herein, we explored a total of 400 adult individuals from seven ethnic groups living in Iran. The lactose tolerance test (LTT) was conducted to discern the LP distribution. We sequenced the relevant genomic region to identify potential variants that are associated with LP.

## Materials and methods

### Population samples

We recruited 400 healthy unrelated volunteers from seven Iranian ethnic groups, including Kurd (*n* = 138), Mazani (*n* = 110), Persian (*n* = 78), Arab (*n* = 26), Lur (*n* = 24), Azeri (*n* = 15), and Gilak (*n* = 9) (Additional file 1: Figure S1). Five-milliliter whole peripheral venous blood samples of the volunteers were collected. The study protocol was along with ethical approval and informed consent in Iran (Babol University of Medical Sciences, MUBABOL.REC.1394.354) and was also approved by the Internal Review Board of Kunming Institute of Zoology, Chinese Academy of Sciences (SMKX2017003).

### Lactose tolerance test

We conducted the lactose tolerance test **(**LTT) in the 400 volunteers as described before [8, 34]. The volunteers were instructed to fast overnight and avoid smoking. The fasting fingertip capillary blood-glucose level at baseline was recorded with the Accu-Chek Advantage glucometer and Accu-Chek Comfort Curve Blood Glucose Test Strips (Roche, Mannheim, Germany) in the next morning. A 50-g lactose powder (Kerry Bio-Science) diluted in 250 mL of water at room temperature was given to each volunteer. The volunteers were requested to stay for the entire test duration (i.e., ~ 1 h). We measured fingertip capillary blood glucose levels in duplicates at 20-min intervals over a 1-h period. The lactase status was classified into three categories on the basis of the maximum rise in glucose level: an individual with a blood glucose level > 1.7 mmol/L was classified as LP; an individual with a blood glucose level < 1.1 mmol/L was classified as lactase non-persistence (LNP); and “lactase intermediate persistent” (LIP) was classified as an individual with a blood glucose level between 1.1 and 1.7 mmol/L.

### PCR and Sanger sequencing

The genomic DNA was extracted from whole blood by a modified salting-out method [35] at Shahid Bahonar University of Kerman, Iran. We amplified and sequenced the 706 bp regulatory region for *LCT* in intron 13 of *MCM6* referring to the previous protocol [36]. The variant − 22018*A was checked by PCR-RFLP [14]. Additionally, we sequenced − 22018*A variant region to confirm the RFLP status in 15 samples. The 683 bp of the control region 1 and 701 bp of the control region 2 for *LCT* were also sequenced in 400 samples [9, 37]. All sequences were checked and aligned by Lasergene (DNAStar Inc., Madison, Wisconsin, USA), and mutations were scored relative to the reference genome sequence (GRCh37/hg19).

### Data analysis

To identify SNPs associated with the LP trait in the Iranian populations, we performed Fisher’s exact test with R statistical software version 3.3.2 (https://www.r-project.org/). We tested the association between LP phenotype and common SNPs (minor allele frequency > 5%) in the 400 individuals. The nucleotide diversity [38] was calculated with DNAsp v. 6.11.01 [39]. We used PHASE v.2.1.1 [40, 41] to phase haplotypes based on 18 SNPs of the regulatory region and its flanking control regions 1 and 2. The haplotypes with fewer than three occurrences were excluded [9]. The median-joining network was constructed with Network v.5.0.0.1 [42].

### Whole-genome resequencing and detection of selective signals

We carried out whole-genome sequencing for 20 Persian individuals from Kerman in Southeastern Iran. The samples were randomly selected for whole-genome resequencing. The sequencing was performed on Illumina HiSeq X Ten. We referred to the GATK Best Practices for the SNP calling [43]. We retrieved the unphased SNP data of the 1 Mb (GRCh37/hg19 chr2:136,108,835-137,108,505) containing the regulatory region for *LCT* from the 20 Persians as well as 107 TSI (Toscani in Italia) in the 1000 Genomes Project [44] for comparison. We phased the data using SHAPEIT2 r727 [45]. For each of the SNPs, the ancestral and derived alleles were determined according to the alignments for six primates (http://ftp.1000genomes.ebi.ac.uk/vol1/ftp/phase1/analysis_results/supporting/ancestral_alignments/). The SNPs with ambiguously ancestral/derived states were discarded. We calculated the extended haplotype homozygosity (EHH) [46] and the integrated haplotype score (iHS) [47] with REHH 2.0 [48] and selscan software [49] (Additional file 1: Methods S1).

## Results

### LP in the Iranian populations

According to the LTT results, LP, LIP, and LNP accounted for 9.5%, 24%, and 66.5% of the total Iranian studied population, respectively (Table 1). The highest frequency of LP was in the Arab population (26.92%), whereas LP was not detected in the Lur population (Table 1). Differences of LP frequency were significant when tabulated by ethnicity (Fisher’s exact test, *P* value = 0.0004), occupation (chi-squared test, *P* value = 0.018), and language (chi-squared test, *P* value = 0.0004).

**Table 1:** Phenotype frequency obtained from lactose tolerance testing among different ethnic groups in Iran

Group	Occupation	Language	Sample Size	LP	LNP	LIP
Kurd	Herder	Indo-European	138 (34.5%)	8 (5.79%)	92 (66.66%)	38 (27.53%)
Mazani	Farmer	Indo-European	110 (27.5%)	9 (8.18%)	77 (70%)	24 (21.81%)
Persian	Herder	Indo-European	78 (19.5%)	12 (15.38%)	52 (66.66%)	14 (17.94%)
Arab	Herder	Afro-Asiatic	26 (6.5%)	7 (26.92%)	13 (50.00%)	6 (23.07%)
Lur	Herder	Indo-European	24 (6.0%)	0 (0.00%)	15 (62.50%)	9 (37.50%)
Gilak	Farmer	Indo-European	9 (2.2%)	1 (11.11%)	6 (66.66%)	2 (22.22%)
Azeri	Farmer	Turkic	15 (3.8%)	1 (6.66%)	11 (73.33%)	3 (20.00%)
Total	–	–	400	38 (9.5%)	266 (66.5%)	96 (24.0%)

### LP variants in the Iranian populations

**Fig. 1:**
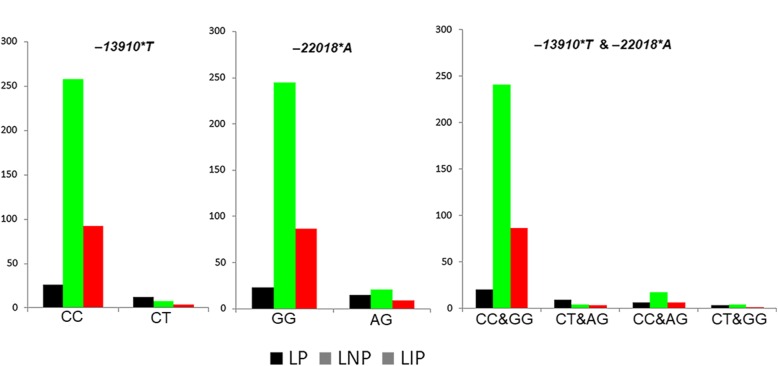
The genotype–phenotype correlation in the merged Iranian population

We identified three LP variants: − 22,018*A, − 13915*G, and − 13910*T, in the Iranian population (Table 2; Additional file 1: Table S1). All three variants were heterozygotes in the carriers. The three variants were also detected in neighboring countries (Additional file 1: Table S2). The − 22018*A allele, the most common variant (11.25%), was detected at the highest frequency in the Arab (19.23%) and the lowest in the Lur (4.16%) populations. The prevalence of − 13910*T was found in most populations (4.16–7.69%) with the exception of the Gilaks. The variant − 13915*G only occurred in one individual from both the Persians (*n* = 78, 1.28%) and the Arabs (*n* = 26, 3.84%), respectively. The co-occurrence of − 22018*A and − 13910*T was observed in all populations except the Lurs and the Gilaks (Additional file 2: Table S5). The variants of − 22018*A (Fisher’s exact test, *P* value = 3.725 × 10^−6^) and − 13910*T (Fisher’s exact test, *P* value = 1.509 × 10^−7^) were significantly associated with LP. Notably, the significant association was also detected between LP and the co-occurrence of − 13910*T and − 22018*A (Fisher’s exact test, *P* value = 1.73 × 10^−7^) (Fig. 1). In a total of 38 LP individuals, nine individuals carried both − 13910*T and − 22018*A. The nucleotide diversity of the regulatory region and the flanking control regions was highest in the LP subpopulation (0.126) and lowest in the LNP subpopulation (0.105) while the LIP individuals showed an intermediate value of 0.112 (Additional file 1: Table S3).

**Table 2:** The distribution of three LP variants in Iranian ethnic groups

Group	Size	*− 22018G/A*		*− 13915 T/G*		*− 13910C/T*	
		GG	GA	TT	TG	CC	CT
Kurd	138 (34.5%)	124 (89.85%)	14 (10.15%)	138 (100%)	0 (0.00%)	128 (92.75%)	10 (7.24%)
Mazani	110 (27.5%)	94 (85.45%)	16 (14.54%)	110 (100%)	0 (0.00%)	106 (96.36%)	4 (3.63%)
Persian	78 (19.5%)	72 (92.30%)	6 (7.69%)	77 (98.72%)	1 (1.28%)	72 (92.30%)	6 (7.69%)
Arab	26 (6.5%)	21 (80.76%)	5 (19.23%)	25 (96.16%)	1 (3.84%)	24 (92.30%)	2 (7.69%)
Lur	24 (6.0%)	23 (95.83%)	1 (4.16%)	24 (100%)	0 (0.00%)	23 (95.83%)	1 (4.16%)
Gilak	9 (2.2%)	8 (88.88%)	1 (11.11%)	9 (100%)	0 (0.00%)	9 (100%)	0 (0.00%)
Azeri	15 (3.7%)	13 (86.66%)	2 (13.33%)	15 (100%)	0 (0.00%)	14 (93.33%)	1 (6.66%)
Total	400	355 (88.75%)	45 (11.25%)	398 (99.50%)	2 (0.50%)	376 (94.02%)	24 (5.98%)

### Haplotype and network analysis

**Fig. 2:**
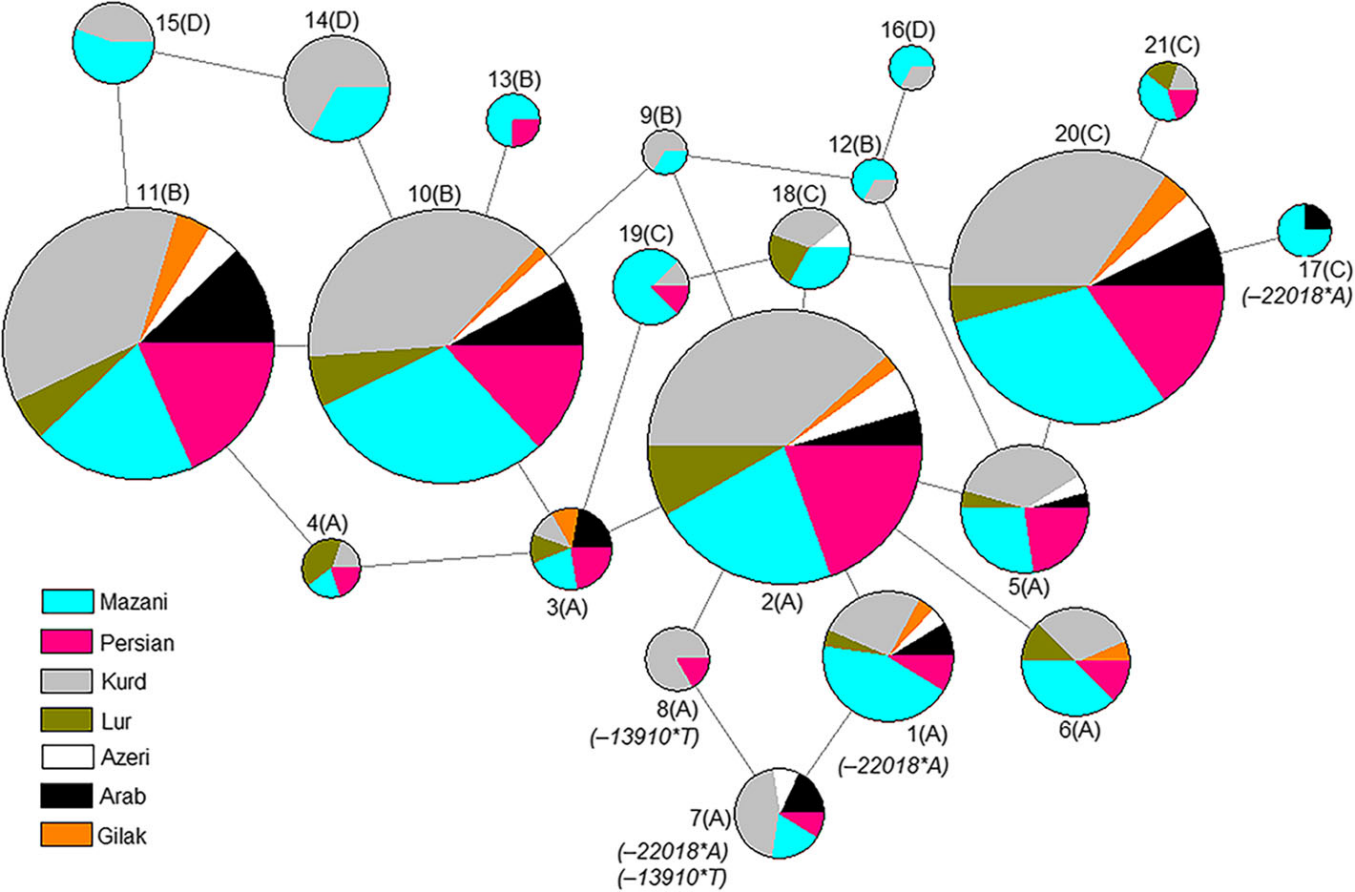
Maximum parsimony neighbor joining network of 18 SNP identified in 400 samples of Iran. Mutations correspond to those in Additional file 1: Figure S2. Each circle represents a haplotype, and circle size is shown proportional to the number of individuals with a given haplotype

We phased the 400 sequences to get 38 haplotypes according to the 18 SNPs of the regulatory region and the flanking control regions (Additional file 1: Table S4). By excluding 17 haplotypes with fewer than three occurrences [9], we plotted 21 major haplotypes in the median-joining network (Fig. 2) according to the nomenclature proposed by Hollox et al. (2001) [37] (Additional file 1: Figure S2). All − 13910*T alleles were observed on haplotype A that is agreement with previous studies [50–52]. The − 22018*A allele was found in haplotypes A and C, suggesting a recombination event or parallel mutation occurrence [31]. Haplotype A with the − 22018*A allele was present in all groups.

### Detection of selective signals of − 13910*T and − 22018*A

**Fig. 3:**
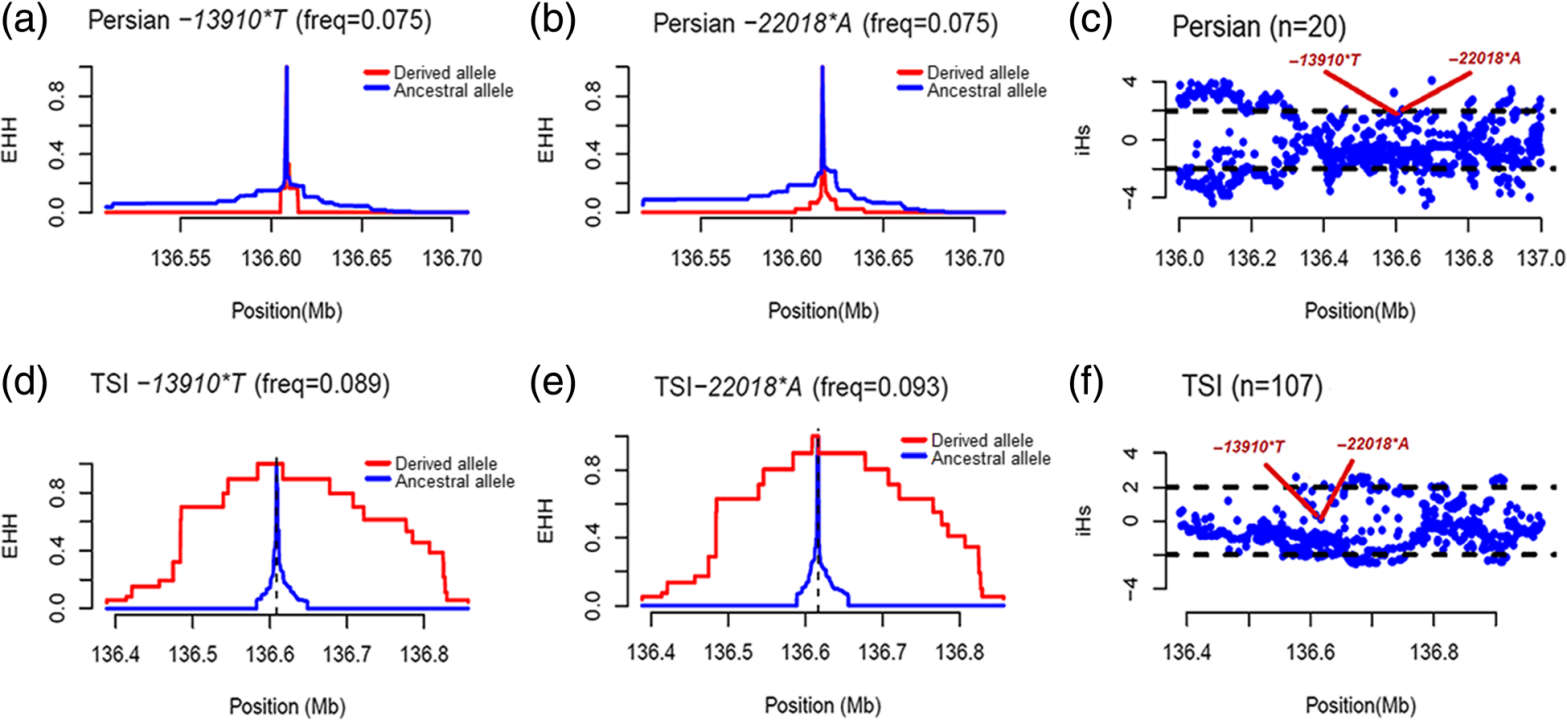
EHH and iHS for the chromosomal positions carrying the − 13910*T and − 22018*A variants. EHH plots in Persian from Kerman, Iran (**a**, **b**) and Toscani in Italy (TSI) (**d**, **e**). Chromosomes containing the derived LP-associated alleles are in red, and those with the ancestral allele are in blue. Chromosomal positions are indicated on the *x*-axes, and the EHH value is indicated on the *y*-axes. The values of iHS are plotted against the genomic position of the SNPs including 1 Mb (chr2:136000000-137000000) at *MCM6* and *LCT* promoter in Persian (**c**) and TSI (**f**). The estimates for − 13910*T and − 22018*A are noted

Because − 13910*T and − 22018*A are associated with LP in the Iranian populations, we tested for indications of a recent selective sweep on both alleles based on the whole genome re-sequencing data of 20 Persians. The alleles − 13910*T and − 22018*A were in complete linkage disequilibrium. The population of TSI presenting the similar characters for those two alleles was used for comparison. The allele frequency was 7.5% (3/40) in Persian (carriers classified as one LP and two LNP) and 9.11% (39/428) in TSI. We found no evidence of EHH for − 13910*T (Fig. 3a) and − 22018*A (Fig. 3b) in the Persians as compared with their ancestral alleles. The haplotypes with − 13910*T (Fig. 3d) and − 22018*A (Fig. 3e) showed EHH in TSI. No significant selective signal (i.e., |iHS| < 2) for − 13910*T and − 22018*A was detected by iHS in the Persians (Fig. 3c) and TSI (Fig. 3f).

## Discussion

Our study depicted the distribution of LP in the Iranian populations. However, the number of recruited subjects for some ethnic groups (Gilak for example) was small. It provided an opportunity to test the culture-historical hypothesis [53] in Iran. The hypothesis suggested that LP had been under historical selection, so that it was popular in populations (e.g., nomads) where milk products served as a substantial dietary component [17]. The prevalence of LP in Iran, with an average of 9.5% in the Iranian populations, was generally lower than that in the populations from Central Asia (14%) [5, 17], Afghanistan (19%), Pakistan (38%), Turkey (30%), Saudi Arabia (81%), and Jordan (76%) [32]. Within our Iranian studied population, the herders presented the higher percentage of LP distribution than the farmers (Table 1), which is consistent with the proposed pattern of the culture-historical hypothesis [53]. In the herders, we found the LP at the highest frequency (29.9%) in the Iranian Arabs. It was compatible with previous studies revealing that the nomadic Arabs had a high frequency of LP [32, 54]. Surprisingly, the Lurs, the traditional pastoral nomads living in the Zagros Mountains [55], were characterized as LIP (37.5%) and LNP (62.5%) but not LP (Table 1). The moderate or even low level of LP frequencies in Iran could be explained by several reasons. The consumption of moderate amounts of fresh milk, averaging just 48.48 kg per person in 2013 (FAOSTAT, http://www.fao.org/faostat/en/#home), may be the main contributing factor. On a deeper level, it may reflect a complex demographic history [56–59] such as admixture among nomadic and secondary populations which was proposed in Central Asia [17].

Our analyses indicated that the − 13910*T and − 22018*A variants were significantly associated with LP in all seven Iranian ethnic groups. However, these associations may not be able to explain all LP in the Iranian populations. In the 39 LP individuals, 20 individuals do not carry − 13910*T and/or − 22018*A (Additional file 2: Table S5). The future analyses based on massive whole genome re-sequencing may have the potential to reveal certain novel LP candidate variants in Iranians. The other thing worth noting is that other non-genetic factors, such as milk allergy [60], gut microbiota [61], and dairy foods [62], should be considered in both LTT and genetic analyses. Moreover, we found no evidence of hard selective sweeps for − 13910*T and − 22018*A in the re-sequenced genomes for 20 Persian individuals (Fig. 3). This result was different from that observed in Europeans [36, 47, 63] and South Asians [6]. Indeed, when considering different demographic scenarios, selection pressure on LP in Iranians was lower than most Europeans [64]. In particular, the occurrence of − 13910*T in two distinguished LP haplotypes was observed in Iranians [31]. Both haplotypes in Iranians were dated within 3000 years [31] that was much later than the dairying practices spread from the West Asia to Europe [65], raising the possibility that the − 13910*T alleles might be introduced into Iran recently. Meanwhile, as similar in Ethiopians [9], the increasing of nucleotide diversity was observed in the Iranian LP individuals (Additional file 1: Table S3). It implied that soft sweep rather than hard sweep played substantial roles in the evolution of LP in Iranian populations.

**Fig. 4:**
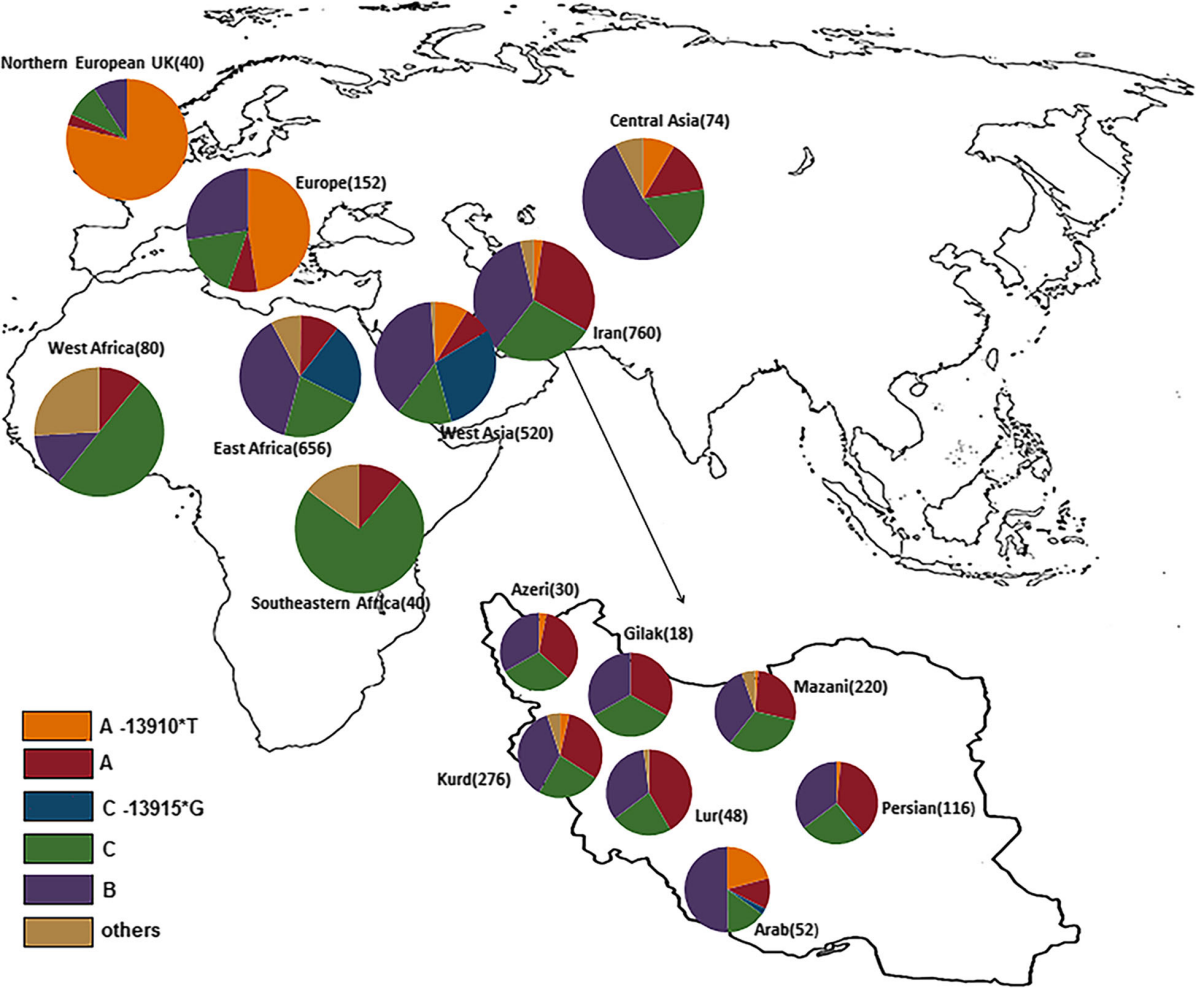
Distribution of *MCM6* haplotypes in different geographic regions. The number of chromosomes is in parentheses

In addition, the distribution of LP variants in Iran would also provide certain clues towards the demographic history for different ethnic groups. The variant − 13915*G showed considerably high frequency in populations of Arabian Peninsula (Additional file 1: Table S2), such as Saudi (0.76) [66], Oman (0.72), and Yemeni (0.54) Arabs [67]. Southern Arabia was proposed to be the origin center of − 13915*G [67] in association with the domestication of Arabian camel (*Camelus dromedarius*) ~ 6000 years ago [7]. The distribution of − 13915*G was also high (10.4–17.6%) in several East African populations [7, 8]; proposing this may be the result of demic diffusion from Arabian Peninsula into East Africa, especially mediated by the spread of Islam in the last 1400 years [68, 69]. The Muslim conquest of Persia led to the fall of the Sasanian Empire (633–654 AD) [70], which also generated lots of cultural changes in Iran. Intriguingly, the Arabic-prevalent − 13915*G was almost absent in most Iranian populations except in one Arab and one Persian (Table 2; Fig. 4). In fact, a relatively high frequency of − 13910*T was detected in the Iranian Arabs (Table 2; Fig. 4). Our results suggested the demic diffusion of − 13915*G from Arabian Peninsula could be minimal in Iran, especially as compared the scenario in East Africa.

## Conclusions

Our results indicate the distribution of LP in seven ethnic groups across the Iranian plateau. Soft selective sweep rather than hard selective sweep played substantial roles in the evolution of LP in Iranian populations. We observe the higher percentage of LP distribution in herders, thus providing evidence for the culture-historical hypothesis [53]. In the future, the integration of archeology [71, 72] and ancient DNA studies [73, 74] may uncover more details about the evolution of LP in Iran, which will also shed novel insights into “the milk revolution” [65] in the Neolithic core zone of West Asia.

## Additional files

Additional file 1: **Table S1.** Allele and genotype frequencies of three LP variants in Iranian ethnic groups. **Table S2.** Allele and genotype frequencies of the neighboring countries of Iran. **Table S3.** Comparison of the levels of nucleotide diversity in lactase persistent, lactase intermediate persistent and lactase nonpersistent measured across the three sequence regions from 400 Iranian Individuals. **Table S4.** The phased 400 sequences into 38 haplotypes based on the 18 SNPs of the regulatory region and the flanking control regions. **Figure S1.** Map of Iran showing approximate locations of the ethnic groups included in the present study. **Figure S2.** Phased Haplotypes for the *LCT* enhancer. Control region 1 and Control region 2, in Intron 9 and 13 of *MCM6* and 1 kb Upstream of *LCT* from 7 ethnic groups in 400 Iranian Individuals. **Method S1.** Whole-Genome Resequencing and Detection of Selective Signals. (DOCX 804 kb)

Additional file 2: **Table S5.** Genotype and phenotype information. (XLSX 53 kb)

## Abbreviations

EHH: Extended haplotype homozygosity
IHS: Integrated haplotype score
LCT: Lactase gene
LIP: Lactase intermediate persistent
LNP: Lactase non-persistence
LP: Lactase persistence
LTT: Lactose tolerance test
MCM6: Minichromosome maintenance complex component 6
PCR: Polymerase chain reaction
RFLP: Restriction fragment length polymorphism
SNPs: Single nucleotide polymorphisms
TSI: Toscani in Italia
