# Correction to: The evolutionary genetics of lactase persistence in seven ethnic groups across the Iranian plateau

**DOI:** 10.1186/s40246-019-0200-z

**Published:** 2019-03-22

**Authors:** Hadi Charati, Min-Sheng Peng, Wei Chen, Xing-Yan Yang, Roghayeh Jabbari Ori, Mohsen Aghajanpour-Mir, Ali Esmailizadeh, Ya-Ping Zhang

**Affiliations:** 1 State Key Laboratory of Genetic Resources and Evolution, Kunming Institute of Zoology, Chinese Academy of Sciences, Kunming, 650223 China; 2 Kunming College of Life Science, University of Chinese Academy of Sciences, Kunming, 650204 China; 3 KIZ-CUHK Joint Laboratory of Bioresources and Molecular Research in Common Diseases, Kunming Institute of Zoology, Chinese Academy of Sciences, Kunming, 650223 China; 4 Biological Big Data College, Yunnan Agricultural University, Kunming, 650201 China; 5 State Key Laboratory for Conservation and Utilization of Bio-resources, Yunnan University, Kunming, 650091 China; 6 Faculty of Agriculture, Shahid Bahonar University of Kerman, Kerman, PB 76169-133 Iran; 7 Department of Genetics, Faculty of Medicine, Babol University of Medical Sciences, Babol, 4719173716 Iran; 8 Department of Medical Genetics, School of Medicine, Tehran University of Medical Sciences, Tehran, 14155-6447 Iran; 9 Center for Excellence in Animal Evolution and Genetics, Chinese Academy of Sciences, Kunming, 650223 China

**Correction to: Hum Genomics (2019) 13:7**

**https://doi.org/10.1186/s40246-019-0195-5**

In the original publication of this article [1], the colors of the Fig. 1 are wrong, and are revised in the updated figure below:

**Fig. 1:**
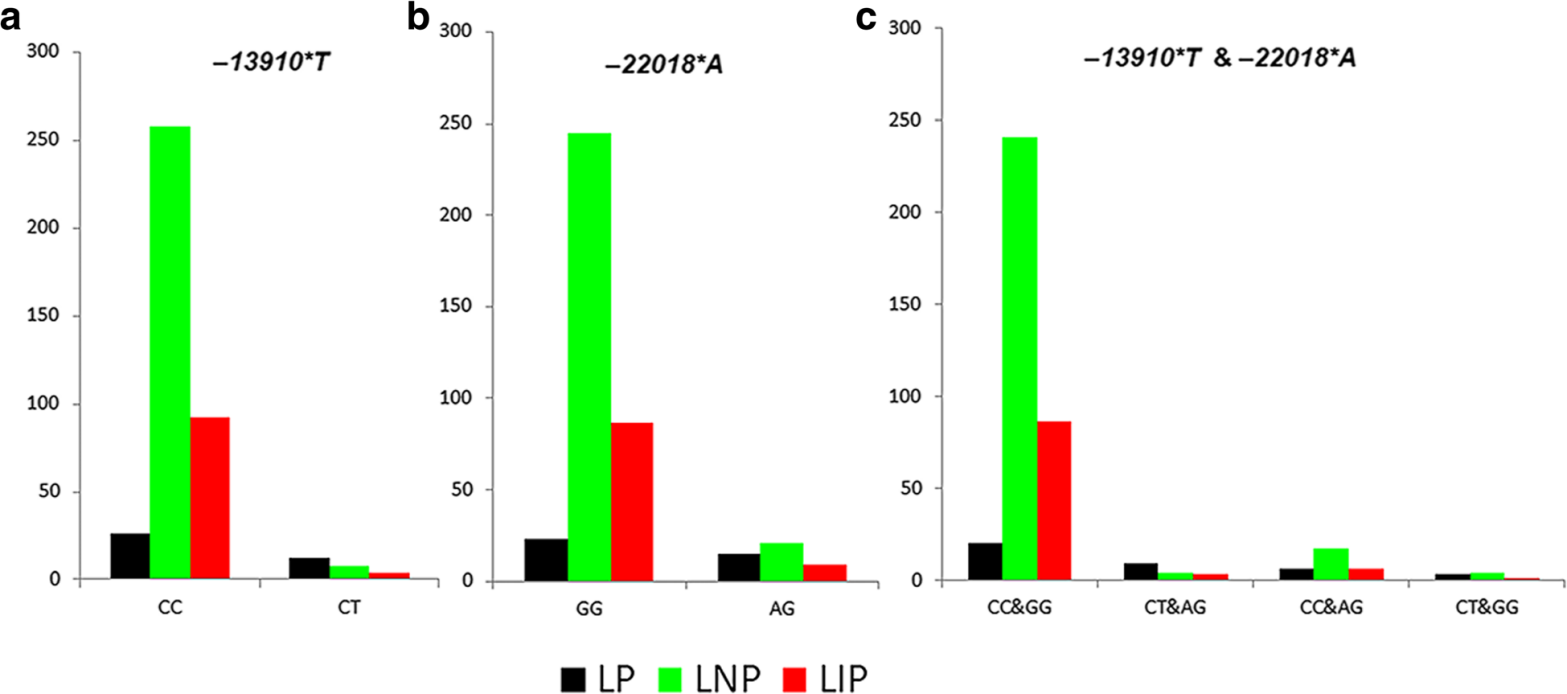
The genotype–phenotype correlation in the merged Iranian population

Also, the number for the award to A. Esmailizadeh during his visit to Chinese Academy of Sciences by Chinese Academy of Sciences President’s International Fellowship Initiative was wrong in the Acknowledgments section, and the right number should be No. 2016VBA050.
