# Correction to: The evolutionary genetics of lactase persistence in seven ethnic groups across the Iranian plateau

**DOI:** 10.1186/s40246-021-00355-y

**Published:** 2021-08-24

**Authors:** Hadi Charati, Min-Sheng Peng, Wei Chen, Xing-Yan Yang, Roghayeh Jabbari Ori, Mohsen Aghajanpour-Mir, Ali Esmailizadeh, Ya-Ping Zhang

**Affiliations:** 1 State Key Laboratory of Genetic Resources and Evolution, Kunming Institute of Zoology, Chinese Academy of Sciences, Kunming, 650223 China; 2 Kunming College of Life Science, University of Chinese Academy of Sciences, Kunming, 650204 China; 3 KIZ-CUHK Joint Laboratory of Bioresources and Molecular Research in Common Diseases, Kunming Institute of Zoology, Chinese Academy of Sciences, Kunming, 650223 China; 4 Biological Big Data College, Yunnan Agricultural University, Kunming, 650201 China; 5 State Key Laboratory for Conservation and Utilization of Bio-Resources, Yunnan University, Kunming, 650091 China; 6 Faculty of Agriculture, Shahid Bahonar University of Kerman, PB 76169-133 Kerman, Iran; 7 Department of Genetics, Faculty of Medicine, Babol University of Medical Sciences, 4719173716 Babol, Iran; 8 Department of Medical Genetics, School of Medicine, Tehran University of Medical Sciences, 14155-6447 Tehran, Iran; 9 Center for Excellence in Animal Evolution and Genetics, Chinese Academy of Sciences, Kunming, 650223 China

## Correction to: Human Genomics (2019) 13:7 10.1186/s40246-019-0195-5

Following publication of the original article [1], the below authorship note is missing in this article due to a typesetting error.

Hadi Charati and Min-Sheng Peng contributed equally to this work.
