# Supplementary material for: The evolutionary genetics of lactase persistence in seven ethnic groups across the Iranian plateau
Source: Hum Genomics. 2019 Feb 11;13:7. doi: 10.1186/s40246-019-0195-5 (PMC6371433; doi:10.1186/s40246-019-0195-5)
Supplement: Supplementary file 1 — Table S1. Allele and genotype frequencies of three LP variants in Iranian ethnic groups. Table S2. Allele and genotype frequencies of the neighboring countries of Iran. Table S3. Comparison of the levels of nucleotide diversity in lactase persistent, lactase intermediate persistent and lactase nonpersistent measured across the three sequence regions from 400 Iranian Individuals. Table S4. The phased 400 sequences into 38 haplotypes based on the 18 SNPs of the regulatory region and the flanking control regions. Figure S1. Map of Iran showing approximate locations of the ethnic groups included in the present study. Figure S2. Phased Haplotypes for the LCT enhancer. Control region 1 and Control region 2, in Intron 9 and 13 of MCM6 and 1 kb Upstream of LCT from 7 ethnic groups in 400 Iranian Individuals. Method S1. Whole-Genome Resequencing and Detection of Selective Signals. (DOCX 804 kb) [file 40246_2019_195_MOESM1_ESM.docx]

**Online resource**

**The evolutionary genetics of lactase persistence in seven ethnic groups across the Iranian plateau**

Hadi Charati . Min-Sheng Peng . Wei Chen . Xing-Yan Yang. Roghayeh Jabbari Ori. Mohsen Aghajanpour-Mir. Ali Esmailizadeh . Ya-Ping Zhang

**Tables**

**Table S1:** Allele and genotype frequencies of three LP variants in Iranian ethnic groups

| **Group** | **Size** | ***–13915T/G*** | | | | | ***–13910C/T*** | | | | | ***–22018G/A*** | | | | |
| --- | --- | --- | --- | --- | --- | --- | --- | --- | --- | --- | --- | --- | --- | --- | --- | --- |
|  |  | **T** | **G** | **TT** | **TG** | **HWE test**  **Chi-square** | **C** | **T** | **CC** | **CT** | **HWE test**  **Chi-square** | **G** | **A** | **GG** | **GA** | **HWE test**  **Chi-square** |
| Kurd | 138 | 1 | 0.00 | 138 (100%) | 0 (0.00%) | 0 | 0.96 | 0.03 | 128 (92.75%) | 10 (7.24%) | 0.19^ns^ | 0.94 | 0.05 | 124 (89.85%) | 14 (10.15%) | 0.39^ns^ |
| Mazani | 110 | 1 | 0.00 | 110 (100%) | 0 (0.00%) | 0 | 0.98 | 0.01 | 106 (96.36%) | 4 (3.63%) | 0.03^ns^ | 0.92 | 0.07 | 94 (85.45%) | 16 (14.54%) | 0.67^ns^ |
| Persian | 78 | 0.99 | 0.00 | 77 (98.72%) | 1 (1.28%) | 0 | 0.96 | 0.03 | 72 (92.30%) | 6 (7.69%) | 0.12^ns^ | 0.96 | 0.03 | 72 (92.30%) | 6 (7.69%) | 0.12^ns^ |
| Arab | 26 | 0.98 | 0.01 | 25 (96.16%) | 1 (3.84%) | 0.01^ns^ | 0.96 | 0.03 | 24 (92.30%) | 2 (7.69%) | 0.04^ns^ | 0.90 | 0.09 | 21 (80.76%) | 5 (19.23%) | 0.29^ns^ |
| Lur | 24 | 1 | 0.00 | 24 (100%) | 0 (0.00%) | 0 | 0.97 | 0.02 | 23 (95.83%) | 1 (4.16%) | 0.01^ns^ | 0.97 | 0.02 | 23 (95.83%) | 1 (4.16%) | 0.01^ns^ |
| Gilak | 9 | 1 | 0.00 | 9 (100%) | 0 (0.00%) | 0 | 1 | 0.00 | 9 (100%) | 0 (0.00%) | 0 | 0.94 | 0.05 | 8 (88.88%) | 1 (11.11%) | 0.03^ns^ |
| Azeri | 15 | 1 | 0.00 | 15 (100%) | 0 (0.00%) | 0 | 0.96 | 0.03 | 14 (93.33%) | 1 (6.66%) | 0.01^ns^ | 0.93 | 0.06 | 13 (86.66%) | 2 (13.33%) | 0.07^ns^ |
| Total | **400** | **0.99** | **0.00** | **398 (99.50%)** | **2 (0.50%)** | **0.00** | **0.97** | **0.03** | **376 (94.02%)** | **24 (5.98%)** | **0.38**^ns^ | **0.94** | **0.05** | **355 (88.75%)** | **45 (11.25%)** | **1.42**^ns^ |

HWE: Hardy–Weinberg equilibrium; ns: not significant at P <0.05.

**Table S2:** Allele and genotype frequencies of the neighboring countries of Iran

| **Country (Group)** | **Size** | ***–13915T/G*** | | | | | | ***–13910C/T*** | | | | | | ***–22018G/A*** | | | | | |
| --- | --- | --- | --- | --- | --- | --- | --- | --- | --- | --- | --- | --- | --- | --- | --- | --- | --- | --- | --- |
|  |  | **T** | **G** | **TT** | **TG** | **GG** | **HWE test**  **Chi-square** | **C** | **T** | **CC** | **CT** | **TT** | **HWE test**  **Chi-square** | **G** | **A** | **GG** | **GA** | **AA** | **HWE test**  **Chi-square** |
| Oman (Arabs of Northern Oman) [1] | 342 | 0.864 | 0.135 | 284(83.04%) | 23(6.72%) | 35(10.23%) | 174.238 | 0.986 | 0.013 | 333(97.368%) | 9(2.631%) | 0(0.00%) | 0.060^ns^ | . | . | . | . | . | . |
| Oman (Omanis of Asian origin) [1] | 96 | 1 | 0 | 96(100%) | 0(0.00%) | 0(0.00%) | 0 | 0.843 | 0.156 | 69(71.875%) | 24(25%) | 3(3.125%) | 0.258^ns^ | . | . | . | . | . | . |
| Oman (Dhofari Arabs of Southern Oman) [1] | 210 | 0.276 | 0.723 | 33(15.714%) | 50(2.380%) | 127(60.476%) | 34.358 | 1 | 0 | 210(100%) | 0(0.00%) | 0(0.00%) | 0 | . | . | . | . | . | . |
| Yemen [1] | 239 | 0.451 | 0.548 | 74(30.962%) | 68(28.451%) | 97(40.585%) | 43.300 | 0.997 | 0.002 | 238(99.581%) | 1(0.418%) | 0(0.00%) | 0.001^ns^ | . | . | . | . | . | . |
| Saudi Arabia [2] | 432 | 0.406 | 0.593 | 100(23.148%) | 151(34.953%) | 181(41.898%) | 32.777 | 0.997 | 0.002 | 430(99.537%) | 2(0.462%) | 0(0.00%) | 0.002^ns^ | . | . | . | . | . | . |
| Syria, Iraq, Lebanon, and Palestine [3] | 19 | 0.894 | 0.105 | 16(84.210%) | 2(10.526%) | 1(5.263%) | 3.698^ns^ | . | . | . | . | . | . | . | . | . | . | . | . |
| Pakistan [4] | 200 | . | . | . | . | . | . | 1 | 0 | . | . | . | . | 0.99 | 0.01 | . | . | . | . |
| Afghanistan [5] | 57 | 1 | 0 | . | . | . | . | 0.80 | 0.19 | . | . | . | . | . | . | . | . | . | . |
| Turkey [6] | 56 | . | . | . | . | . | . | . | . | . | . | . | . | 0.90 | 0.10 | . | . | . | . |
| Kuwait [6] | 15 | . | . | . | . | . | . | . | . | . | . | . | . | 0.93 | 0.06 | . | . | . | . |

HWE: Hardy–Weinberg equilibrium; ns: not significant at P <0.05.

**.** , not determined.

**Table S3:** Comparison of the levels of nucleotide diversity in lactase persistent, lactase intermediate persistent and lactase nonpersistent measured across the three sequence regions from 400 Iranian Individuals

| **Sequence Regions** | **LTT** | **Arab** | **Azeri** | **Gilak** | **Kurd** | **Lur** | **Mazani** | **Persian** | **total** |
| --- | --- | --- | --- | --- | --- | --- | --- | --- | --- |
| Control region 1 | LP | 0.066 | - | - | 0.085 | - | 0.130 | 0.078 | 0.095 |
|  | LIP | 0.173 | 0.066 | 0.150 | 0.103 | 0.122 | 0.113 | 0.102 | 0.110 |
|  | LNP | 0.098 | 0.118 | 0.153 | 0.103 | 0.106 | 0.089 | 0.111 | 0.101 |
| Control region 1  &  Enhancer  &  Control region 2 | LP | 0.111 | - | - | 0.121 | - | 0.162 | 0.093 | 0.126 |
|  | LIP | 0.159 | 0.111 | 0.138 | 0.104 | 0.112 | 0.118 | 0.110 | 0.112 |
|  | LNP | 0.099 | 0.118 | 0.124 | 0.117 | 0.103 | 0.099 | 0.109 | 0.105 |
| Control region 2 | LP | 0.133 | - | - | 0.164 | - | 0.216 | 0.100 | 0.156 |
|  | LIP | 0.226 | 0.133 | 0.200 | 0.149 | 0.161 | 0.169 | 0.152 | 0.158 |
|  | LNP | 0.130 | 0.170 | 0.166 | 0.154 | 0.133 | 0.157 | 0.152 | 0.153 |

**Table S4:** The phased 400 sequences into 38 haplotypes based on the 18 SNPs of the regulatory region and the flanking control regions

| **INDEX** | **HAPLOTYPE** | **Number of chromosomes** | **freq** |
| --- | --- | --- | --- |
| 1 | CAGACCTAGTAACGGTTT | 24 | 0.030 |
| 2 | CAGACCTGGTAACGGTTT | 165 | 0.206 |
| 3 | CAGACCTGGTAACGCTTT | 11 | 0.013 |
| 4 | CAGACCTGGTAACGCTTG | 5 | 0.006 |
| 5 | CAGACCTGGTAACCGTTT | 2 | 0.002 |
| 6 | CAGACCTGGTAGCGGTTT | 23 | 0.028 |
| 7 | CAGACCTGGTAGCGGTGT | 1 | 0.001 |
| 8 | CAGACCTGGTAGTGGTTT | 1 | 0.001 |
| 9 | CAGACCTGGTTACGGTTT | 2 | 0.002 |
| 10 | CAGACCTGCTAACGGTTT | 16 | 0.020 |
| 11 | CAGACTTAGTAACGGTTT | 14 | 0.017 |
| 12 | CAGACTTAGTAACGCTTT | 1 | 0.001 |
| 13 | CAGACTTGGTAACGGTTT | 6 | 0.007 |
| 14 | CAGATCTAGTAACGCTTG | 2 | 0.002 |
| 15 | CAGATCTGGTAACGGTTT | 3 | 0.003 |
| 16 | CAGATCTGGTAACGGTTG | 1 | 0.001 |
| 17 | CAGATCTGGTAACGCTTT | 164 | 0.205 |
| 18 | CAGATCTGGTAACGCTTG | 98 | 0.122 |
| 19 | CAGATCTGGTAGCGGTTT | 3 | 0.003 |
| 20 | CAGATCTGGTAGCGCTTG | 1 | 0.001 |
| 21 | CAGATCTGGAAACGCTTT | 2 | 0.002 |
| 22 | CAGATCTGGAAACGCTTG | 1 | 0.001 |
| 23 | CAGATTTGGTAACGCTTT | 2 | 0.002 |
| 24 | CAGATTTGGTAACGCTTG | 1 | 0.001 |
| 25 | CAGGTCTGGTAACGCTTT | 4 | 0.005 |
| 26 | CAGGTCTGGTAGCGCTTT | 1 | 0.001 |
| 27 | CAAATCTGGTAACGCTTT | 17 | 0.021 |
| 28 | CAAATCTGGTAACGCTTG | 9 | 0.011 |
| 29 | CAAATCTGGTAGCGGTTT | 3 | 0.003 |
| 30 | CAAATCTGGAAACGCTTT | 1 | 0.001 |
| 31 | CGGACCTAGTAGCGGTTT | 4 | 0.005 |
| 32 | CGGACCTGGTAACGGTTT | 10 | 0.012 |
| 33 | CGGACCTGGTAACGCTTT | 8 | 0.010 |
| 34 | CGGACCTGGTAGCGGTTT | 184 | 0.230 |
| 35 | CGGACCTGGTAGCGGCTT | 5 | 0.006 |
| 36 | CGGACCTGCTAACGGTTT | 2 | 0.002 |
| 37 | CGGACCGGGTAGCGGTTT | 2 | 0.002 |
| 38 | GAGATCTGGTAGCGCTTT | 1 | 0.001 |

**Figures**

**Figure S1.** Map of Iran showing approximate locations of the ethnic groups included in the present study

**
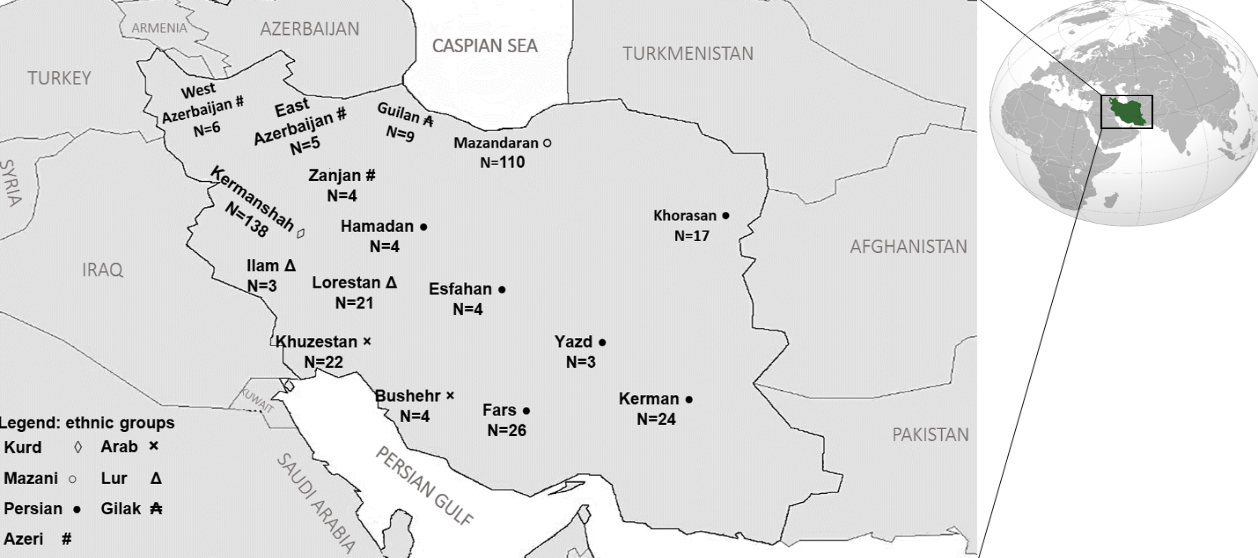
**

**Figure S2. Phased Haplotypes for the *LCT* enhancer ,Control region 1 and Control region 2, in Intron 9 and 13 of *MCM6* and 1 kb Upstream of *LCT* from 7 ethnic groups in 400 Iranian Individuals**. The SNP sites are numbered and refered to the following chromosomal positions relative to *LCT*; 1, *–30376 *G*; 2, *–30355*G*; 3*, –30252*C*; 4, *–30210*C*; 5, *–30205*C*; 6, *–30183*T*; 7, *–30182*G*; 8, *–30160*T*; 9, –*30138*A*; 10, *–29949*C*; 11, *–22018*A*; 12, *–13915*G*; 13, *–13910*T*; 14, *–958*T*; 15, *–957*G*; 16*,–875 *A*; 17, *–678**G; 18, *–668*G*. N: number of chromosomes, ■: the new allele, □: the ancestral allele. Haplotypes occurring less than three times are not shown. The lettered haplotypes that are shown refer to the haplotypes previously reported by Hollox et al. (2001) [7]. Those that are shown in bold are the most probable equivalents [1].


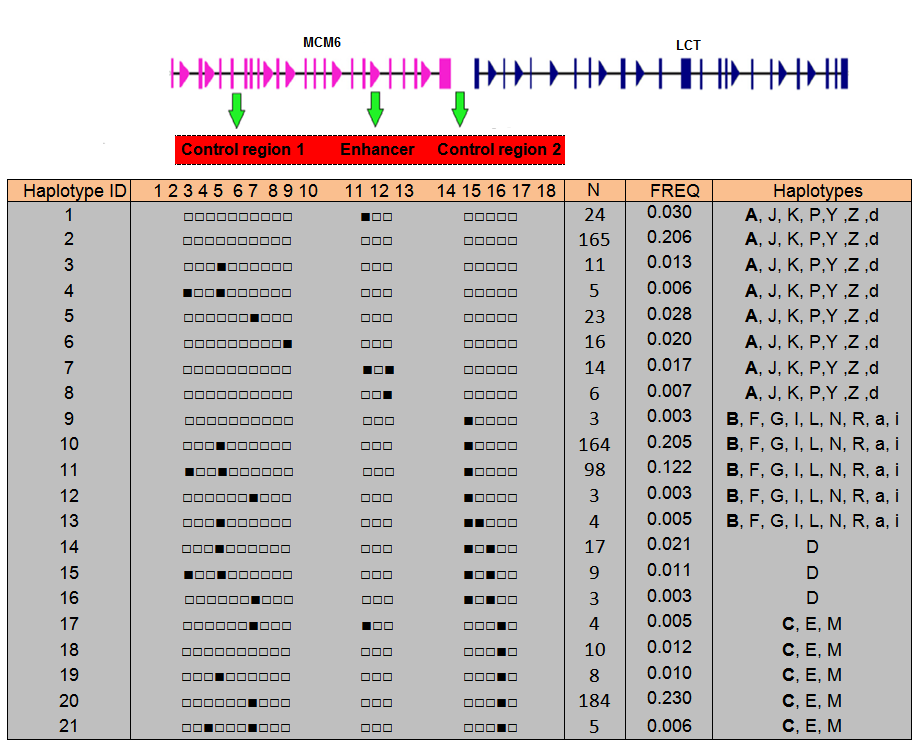


**Methods**

**Method S1. Whole-Genome Resequencing and Detection of Selective Signals.**

We carried out whole genome sequencing for 20 Persian individuals from Kerman in eastern Iran. The samples were randomly selected. The sequencing was performed on illumina HiSeq X Ten. The experiment was designed in order to obtain 150 bp pair-end reads and an average coverage of 30X. BWA v0.7 [8] was used to map reads against the human reference genome (GRCh37/hg19). We then used the SAMtools v1.1 [9] to sort and index the raw alignments and Picard v1.98 (http://broadinstitute.github.io/picard/) to mark duplicated reads. SNP calling referred to the GATK (version 3.4) Best Practices [10] with the HaplotypeCaller program. The called genotypes were output to vcf format and filtered the variants with a Phred quality score < 30. Recalibration file is created by the VariantRecalibrator for the SNPs and the using a tranche sensitivity of 99% to filter out likely false variants based on variant quality score. The markers on mitochondrial DNA and sex chromosomes were disregarded. Finally, we performed the quality control on called genotypes with PLINK v1.07 (Purcell S. PLINK. Version 1.07. http://pngu.mgh.harvard.edu/purcell/plink/) [11]. We excluded SNPs with the call rate less than 95%, minor allele frequency under 0.05, and significant departure from Hardy-Weinberg equilibrium (P < 1 × 10^-4^) to obtain autosomal SNPs for subsequent analyses. We retrieved the unphased SNP data of the 1 Mb (GRCh37/hg19 chr2:136,108,835-137,108,505) containing the regulatory region for *LCT* from the 20 Persians as well as 107 TSI (Toscani in Italia) in the 1000 Genomes Project [12] for comparison. We phased the data using SHAPEIT2 v2. r727 [13] with default parameters referring to the genetic map of HapMap phase 2 and the panel of 1000 Genomes Project Phase 3. For each of SNPs, the ancestral and derived alleles were determined according to the alignments for six primates (http://ftp.1000genomes.ebi.ac.uk/vol1/ftp/phase1/analysis_results/supporting/ancestral_alignments/). The SNPs with ambiguously ancestral/derived states were discarded. We calculated the extended of haplotype homozygosity (EHH) [14] and the integrated haplotype score (iHS) [15] with REHH 2.0 [16] and selscan software [17] to identify signatures of recent positive selection on the basis of long-range LD patterns on chromosomes containing the LP-associated SNPs. The EHH was ran as “--ehh-win 100000 --cutoff 0.05 --gap-scale 20000 --maf 0.05 --max-gap 200000”; and the iHS was run as “--pi-win 100 --cutoff 0.05 --gap-scale 20000 --maf 0.05 --max-gap 200000”.

**References**

1. Al-Abri AR, Al-Rawas O, Al-Yahyaee S, Al-Habori M, Al-Zubairi AS, Bayoumi R. Distribution of the lactase persistence-associated variant alleles –13910* T and –13915* G among the people of Oman and Yemen. Hum Biol. 2012;84:271-286.
2. Imtiaz F, Savilahti E, Sarnesto A, Trabzuni D, Al-Kahtani K, Kagevi I, et al. The T/G –13915 variant upstream of the lactase gene (LCT) is the founder allele of lactase persistence in an urban Saudi population. J Med Genet. 2007;44:e89.
3. Enattah NS, Jensen TG, Nielsen M, Lewinski R, Kuokkanen M, Rasinpera H, et al. Independent introduction of two lactase persistence alleles into human populations reflects different history of adaptation to milk culture. Am J Hum Genet. 2008;82:57-72.
4. Bersaglieri T, Sabeti PC, Patterson N, Vanderploeg T, Schaffner SF, Drake JA, et al. Genetic signatures of strong recent positive selection at the lactase gene. Am J Hum Genet. 2004;74:1111-20.
5. Itan Y, Jones BL, Ingram CJ, Swallow DM, Thomas MG. A worldwide correlation of lactase persistence phenotype and genotypes. BMC Evol Biol. 2010;10:36.
6. ALFRED: the allele frequency database . https://alfred.med.yale.edu/alfred/index.asp.
7. Hollox EJ, Poulter M, Zvarik M, Ferak V, Krause A, Jenkins T, et al. Lactase haplotype diversity in the Old World. Am J Hum Genet. 2001;68:160-72.
8. Li H, Durbin R. Fast and accurate long-read alignment with Burrows-Wheeler transform. Bioinformatics. 2010; 26:589-95.
9. Li H, Handsaker B, Wysoker A, Fennell T, Ruan J, Homer N, et al. The Sequence Alignment/Map format and SAMtools. Bioinformatics. 2009;25:2078-9.
10. DePristo MA, Banks E, Poplin R, Garimella KV, Maguire JR, Hartl C, et al. A framework for variation discovery and genotyping using next-generation DNA sequencing data. Nat Genet. 2011;43:491-98.
11. Purcell S, Neale B, Todd-Brown K, Thomas L, Ferreira MAR, Bender D, et al. PLINK: a tool set for whole-genome association and population-based linkage analyses. Am J Hum Genet. 2007;81:559–575
12. Auton A, Brooks LD, Durbin RM, Garrison EP, Kang HM, Korbel JO, et al. A global reference for human genetic variation. Nature. 2015;526:68-74.
13. Delaneau O, Zagury JF, Marchini J. Improved whole-chromosome phasing for disease and population genetic studies. Nat Methods. 2013;10:5-6.
14. Sabeti PC, Reich DE, Higgins JM, Levine HZP, Richter DJ, Schaffner SF, et al. Detecting recent positive selection in the human genome from haplotype structure. Nature. 2002;419:832-37.
15. Voight BF, Kudaravalli S, Wen X, Pritchard JK. A map of recent positive selection in the human genome. PLoS Biol. 2006;4:e72.
16. Gautier M, Klassmann A, Vitalis R. rehh 2.0: a reimplementation of the R package rehh to detect positive selection from haplotype structure. Mol Ecol Resour. 2017;17:78-90.
17. Szpiech ZA, Hernandez RD. Selscan: an efficient multithreaded program to perform EHH-based scans for positive selection. Mol Biol Evol. 2014;31:2824–2827.
